# Supplementary material for: A machine learning-based SNP-set analysis approach for identifying disease-associated susceptibility loci
Source: Sci Rep. 2022 Sep 22;12:15817. doi: 10.1038/s41598-022-19708-1 (PMC9499949; doi:10.1038/s41598-022-19708-1)

Appendix

Table A. SNP-sets significantly associated with HBsAg seroclearance (*p-value* < 0.0005)

SNP-set	List of SNPs	test-statistic	p-value*
1	**rs6749972**, **rs1558599**, **rs11891860**, **rs17584600**	0.1760646465	4.00E-04
2	**rs6462008**, **rs6947275**, **rs6462003**	0.1263838384	2.00E-04
3	**rs10838245**, **rs1809862**, **rs10769023**, **rs2017434**, **rs2047456**, **rs7945342**, **rs872751**	0.2967616162	3.00E-04
4	rs2399971, **rs10508462**, **rs2153442**, **rs4748035**	0.241240404	2.00E-04
5	rs6731235, rs199703414, rs16829541, rs1485096, rs2341849	0.2778494949	2.00E-04

6	rs2119977, rs6826277, **rs11931577**	0.181420202	1.00E-04
7	rs35689347, **rs8037510**, **rs2173091**	0.1386888889	3.00E-04
8	rs28365850, rs62625038, rs17102970	0.1339040404	4.00E-04
9	rs59659073, rs10754962, rs2380525	0.1153363636	4.00E-04
10	rs200957040, rs1499880, rs4857702	0.1084454545	4.00E-04
11	rs12644266, rs13130260, rs6815422	0.1604717172	1.00E-04

*p-values were obtained from 10000 permutations. SNPs in boldface are those that obtained a p-value less than 10^{-4} in Kim et al's GWAS.

Figure A. SNP scoring diagram, where *i* is the counter for each fold in the LOOCV

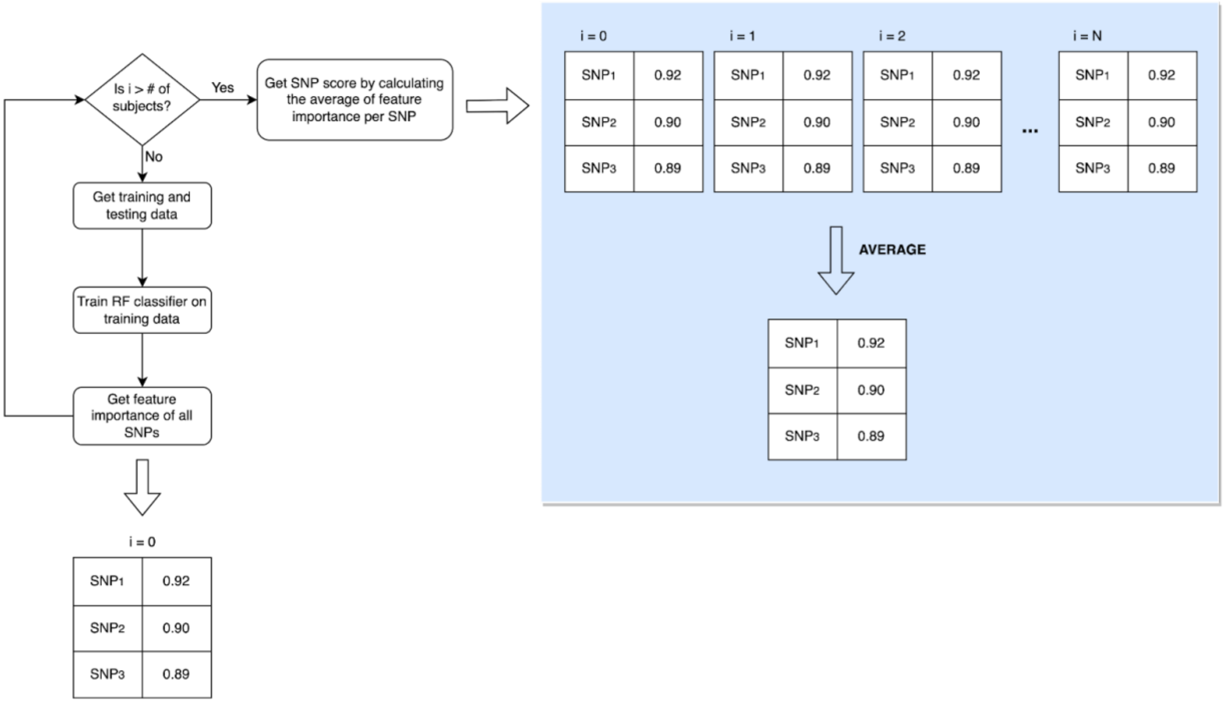

Supplement: Supplementary file 1 — Supplementary Information. [file 41598_2022_19708_MOESM1_ESM.pdf]
